# Medical Journals of the West

**Published:** 1838

**Authors:** 


### Art. V.—Medical Journals in the West.

1. A new number (the 41st) of the *Transylvania Journal*, has lately made its appearance, edited by Professors Cross and Peter. It was due last January. The revolutions in the school from which it emanates will account for the delay. This is the oldest medical periodical, except our own, in the West. Each Quarterly number consists of 200 pages making a volume of 800—price $5.

2. The *Louisville Journal of Medicine and Surgery*, is the title of a new periodical, the first number of which, was issued early in the present year. It is edited by Professors Yandell and Miller of the Louisville Medical Institute, and Dr. Theodore S. Bell.—Prof. Caldwell is a large contributor to the only number which has yet appeared. Each number of this work is to extend to 250 pages, equal 1000 annually, at $5.

3. Our own *Western*, makes a volume of 660 pages, for $3.

If we make a comparison of the prices and number of pages of these periodicals, we have the following result:

One dollar pays for 160 pages of the *Transylvania*, 200 pages of the *Louisville*, and 220 pages of the *Western*. It appears, then, that for the same amount of money, the Lexington gives the least, and our own the most matter. Let those who complain of our dulness recollect this fact. We make up in quantity for our relative deficiency in quality.

Should the three Journals now in the Valley of the Mississippi be sustained, it will be creditable to the profession of the West. They extend to 2,500 pages annually, of which we suppose, at least 3000 copies are printed and distributed. Each of them emanates from a medical school, and of course in its special interest. Having such an origin and object, they are likely, sooner or later, fall into conflict; but this would not, *ex necessitate*, do any harm to themselves, the institutions which put them forth, or the profession. A war among periodicals, prosecuted according to the principles of truth and honor, would but raise their temperature—as the artillery of armies, is heated in battle—an effect of which the public are not likely to complain. Repetition, stereotypism, and dulness, are the besetting sins of periodicals devoted to medical science (including our own) and almost *any thing* which might ward them would be tolerated. D.

